# Association of circulating tumor cells and *IMP3* expression with metastasis of osteosarcoma

**DOI:** 10.3389/fonc.2023.819357

**Published:** 2023-02-23

**Authors:** Shuangwu Dai, Xinxin Shao, Qingzhu Wei, Shaohua Du, Changhe Hou, Haomiao Li, Dadi Jin

**Affiliations:** ^1^ Department of Musculoskeletal Oncology, Center for Orthopaedic Surgery, The Third Affiliated Hospital of Southern Medical University, Guangzhou, China; ^2^ Department of Anesthesiology, The First Affiliated Hospital of Sun Yat-Sen University, Guangzhou, China

**Keywords:** circulating tumor cells, EMT, osteosarcoma, IMP3, metastasis

## Abstract

**Background:**

Circulating tumor cells (CTCs) have been identified as a prognostic biomarker of tumors such as breast cancer and nasopharyngeal carcinoma, because they are obtained through a simple and noninvasive blood draw or liquid biopsy, but its clinical significance in osteosarcoma is still unclear. In this study, we analyzed the relationship between CTCs and clinicopathological features and discussed whether CTCs could be used as a biomarker for metastasis in osteosarcoma.

**Methods:**

We enrolled 50 osteosarcoma patients with Enneking Stage IIB and Stage III and detected CTCs in 5 ml of peripheral blood samples collected from patients using the Canpatrol^®^ CTC detection platform. Subsequently, multiplex RNA *in situ* hybridization (RNA-ISH) based on various molecular markers was performed to identify and classify CTCs. The relationships between clinical pathological features and CTC counts, subtypes (epithelial type, E type; hybrid epithelial/mesenchymal type, H type; mesenchymal type, M type), and insulin-like growth factor mRNA-binding protein 3 (*IMP3*) expression in CTCs were analyzed.

**Results:**

CTCs were detected in 86% (43/50) of the osteosarcoma patients. The CTC counts, especially the total CTCs and H-type CTCs, signifcantly differed between Enneking Stage IIB and Stage III patients (*P* < 0.05). No significant differences were observed between the CTC count or type and other clinicopathological features (*P* > 0.05). There were significant differences in the expression of *IMP3* in different types of CTCs, and the *IMP3* positive rates in E/H/M type CTCs were 38.4, 65.6, and 62.0%, respectively (*P* < 0.001). Receiver operating characteristic (ROC) curve analysis showed that *IMP3*-positive CTC count had the best performance for diagnostic metastasis, with the largest area under the curve of 0.873 and cutoff value of four cells/5ml blood (sensitivity = 87.5%; specificity = 82.4%). Serial CTC monitoring in one patient suggested that total CTCs and H-type CTCs were associated with disease progression.

**Conclusion:**

This study demonstrates that the CTCs, especially the *IMP3*-positive CTCs and H/M-type CTCs, are related to the metastasis of osteosarcoma.

## Introduction

Osteosarcoma is the most common primary malignant bone tumor, arising in children and adolescent (1). Recurrence and metastasis are the most important factors leading to death in patients with osteosarcoma (2). A large proportion of patients with non-metastatic diseases will experience disease recurrence or metastasis after surgical treatment, which will ultimately result in mortality (3). It is well known that the osteosarcoma often spread *via* the hematogenous route to the lung. When patients are determined to have metastasis, the prognosis is very poor (4). Hence, there is a great need for biomarkers that can accurately evaluate the therapeutic efficacy and predict the recurrence and metastasis of osteosarcoma. Based on the metastatic characteristics of osteosarcoma, the exploration of blood circulation markers seems to be a very promising idea.

Circulating tumor cells (CTCs), which release into the blood circulation from primary tumor, are considered as viable metastatic precursors that can initiate clonal metastases. The value of CTCs in prognosis prediction and metastasis monitoring has been confirmed in a variety of tumors such as breast cancer, pancreatic cancer, nasopharyngeal carcinoma, prostate cancer, and hepatocellular carcinoma (5–11). The burden of CTCs is strongly associated with cancer prognosis in several cancer types (12). In addition, a growing number of studies have found that CTCs are not homogeneous populations of cancer cells including the spectrum of epithelial to mesenchymal phenotypes. Epithelial CTCs (E-type CTCs) are only positive for epithelial-related markers (EpCAM and cytokeratins 8/18/19). Mesenchymal CTCs (M-type CTCs) only express mesenchymal markers (vimentin and twist). Hybrid epithelial/mesenchymal CTCs (H-type CTCs) simultaneously express epithelial and mesenchymal markers (13–15). CTCs play an important role in tumor invasion and metastasis through the process of epithelial mesenchymal transition (EMT), or its reverse process termed the mesenchymal to epithelial transition (MET) in single cells. Tumor cells undergoing EMT display enhanced ability to enter the circulation eventually establish metastases in MET (16). Osteosarcoma is a malignant osteogenic tumor originated from mesenchymal cells, and hematogenous spread is the most common mechanism of metastasis. Several studies in osteosarcoma showed a strong correlation between CTCs count and tumor stage or prognosis (17–20). However, the cutoff value of CTC-assisted diagnosis of tumor metastasis is not clear so far.

Insulin-like growth factor mRNA-binding protein 3 (*IMP3*), also known as IGF2BP3, is highly expressed in various cancers and can regulate the expression of various genes at the posttranscriptional level (21, 22). *IMP3* plays an important role in the occurrence, development, and prognosis of osteosarcoma, and the expression of *IMP3* is considered to be an important feature of osteosarcoma (23). It has been shown that *IMP3* is a key regulator of stem-like tumorigenic characteristics in osteosarcoma cells, and its expression is found to confer malignant properties such as anchorage-independent growth, loss of contact inhibition, and escape from anoikic *in vitro*. Notably, in these studies, immunohistochemical method was used to detect the *IMP3* expression in osteosarcoma tumor tissues, and *IMP3* protein is expressed in almost all osteosarcoma tissues (24–26). However, no study explored the expression of *IMP3* in CTCs. In addition, the relationship between *IMP3* expression and the processing of EMT in osteosarcoma CTCs is unclear.

Therefore, here, we used an advanced CTC enrichment identification technology Canpatrol^®^ System to detect CTCs as well as *IMP3* expression in CTCs of osteosarcoma patients (27) and analyzed the relationship between CTCs and the clinicopathological characteristics of osteosarcoma patients to explore whether CTCs could be used as a biomarker for prognosis or metastasis prediction of osteosarcoma. We also evaluated the relationships between *IMP3* expression in CTCs and EMT phenotypes or tumor metastasis, to investigate the clinical significance of CTCs and *IMP3* expression in osteosarcoma.

## Material and methods

### Patients and blood samples collection

**Figure 1:**
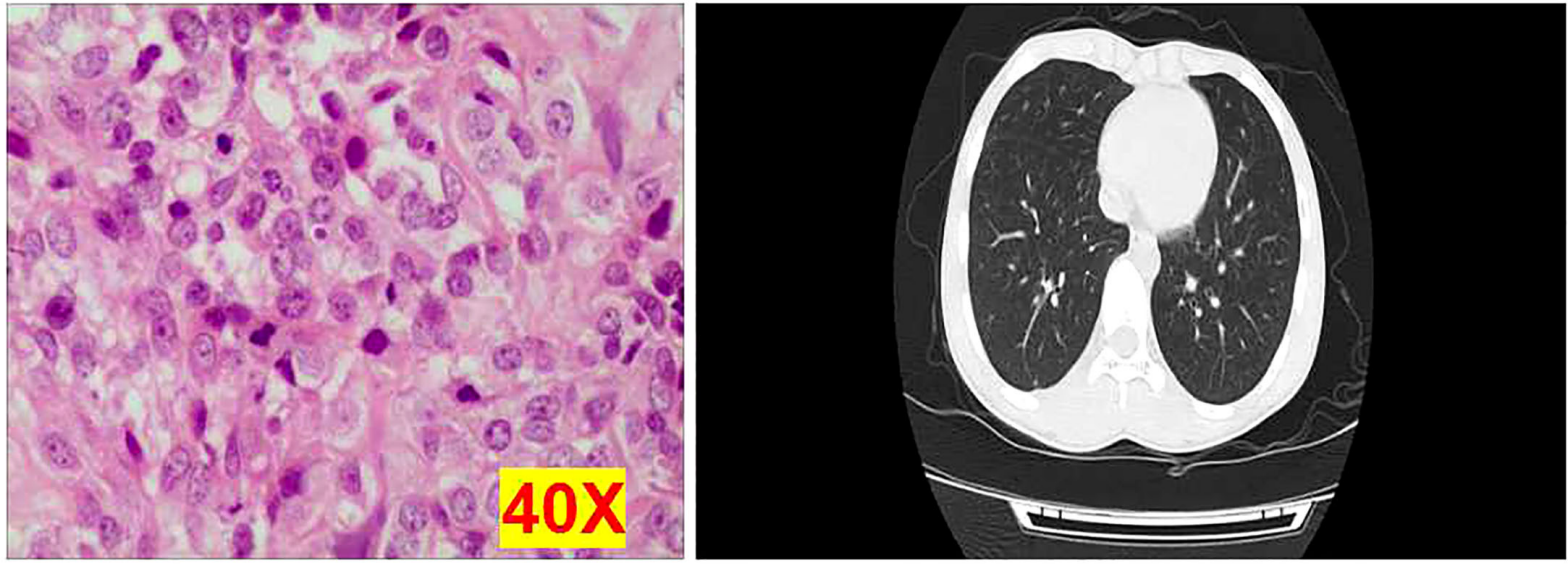
Representative images of osteosarcoma tissues using hematoxylin and eosin staining and computed tomography examination. Each experiment was repeated for three times, and each group had four replicates.

From January 2015 to January 2020, a total of 50 osteosarcoma patients including 16 metastatic osteosarcoma patients and 34 non-metastatic osteosarcoma patients were recruited in this study, and their diagnosis was confirmed by pathological examination and computed tomography (CT) (**Figure 1**) at the Third Affiliated Hospital of Southern Medical University. This study was approved by the Third Affiliated Hospital of Southern Medical University Clinical Trial Ethics Committee, China (No. 2021-lunshen-052). All subjects over 18 years signed an informed consent form, and written informed consents were also signed by a parent or legal guardian for participants under the age of 18 years. None of the patients had received any treatment before. Clinical characters were collected, namely, age, gender, Enneking Stage, tumor location, and other clinicopathological features. In addition, five healthy donors were enrolled as the negative controls.

To avoid cell contamination caused by skin vein puncture, the first 2 ml of peripheral blood was discarded, and then 5 ml of blood was collected into an Ethylenediaminetetraacetic acid (EDTA) tube. Canpatrol System (SurExam Biotech, Guangzhou, China) was used for analysis within 4h after blood collection.

### Isolation of CTCs from peripheral blood

Previously reported Canpatrol platform was used to enrich and identify CTCs from blood (27). In the separation process of CTCs, red blood cells in blood samples were firstly removed with red blood cell lysis buffer, and then leukocytes were removed with a membrane filtration system with the pore size of 8 µm. The required filtration system consisted of a filtration tube containing the membrane (SurExam, Guangzhou, China), a manifold vacuum plate with valve settings (SurExam, Guangzhou, China), an E-Z 96 vacuum manifold (Omega, Norcross, GA, USA), and a vacuum pump (Auto Science, Tianjin, China). The working pressure of the pump valve should be at least 0.08 MPa. Each experiment was repeated for three times, and each group had four replicates.

### Identification of CTCs and detection of *IMP3 *messenger RNA expression level by RNA *in situ* hybridization

CTCs were identified by RNA-ISH method. The CTC subsets were identified by EMT markers according to differential expressions of cytokeratins 8/18/19, epithelial cell adhesion molecule (EpCAM), vimentin, twist, and the leukocyte marker CD45.

*IMP3* mRNA expression level in CTCs was also detected by RNA-ISH assay when EMT biomarker probes were hybridized. The capture probe specific for *IMP3* mRNA was used to capture *IMP3* mRNA, followed by conjugation to the branched DNA (b-DNA) signal amplification probes to create a branched structure. Finally, the labeled probes conjugated with a fluorescent dye were hybridized to the b-DNA sequence. A fluorescence microscope (Olympus BX53, Tokyo, Japan) was used to detect the images. These capture probes were designed based on NCBI database, and these probes were synthesized by Invitrogen (Invitrogen, Shanghai, China). The probe sequence details are shown in **Table 1**.

**Table 1:** EMT typing probe and IMP3 probe.

Genes	Sequence (5′-3′)
*CD45*	TCGCAATTCTTATGCGACTC TGTCATGGAGACAGTCATGT GTATTTCCAGCTTCAACTTC CCATCAATATAGCTGGCATT TTGTGCAGCAATGTATTTCC TACTTGAACCATCAGGCATC
*EpCAM*	TGGTGCTCGTTGATGAGTCA AGCCAGCTTTGAGCAAATGA AAAGCCCATCATTGTTCTGG CTCTCATCGCAGTCAGGATC TCCTTGTCTGTTCTTCTGAC CTCAGAGCAGGTTATTTCAG
*CK8*	CGTACCTTGTCTATGAAGGA ACTTGGTCTCCAGCATCTTG CCTAAGGTTGTTGATGTAGC CTGAGGAAGTTGATCTCGTC CAGATGTGTCCGAGATCTGG TGACCTCAGCAATGATGCTG
*CK18*	AGAAAGGACAGGACTCAGGC GAGTGGTGAAGCTCATGCTG TCAGGTCCTCGATGATCTTG CAATCTGCAGAACGATGCGG AAGTCATCAGCAGCAAGACG CTGCAGTCGTGTGATATTGG
*CK19*	AAGTCATCTGCAGCCAGACG CTGTTCCGTCTCAAACTTGG TTCTTCTTCAGGTAGGCCAG CTCAGCGTACTGATTTCCTC CTGTAGGAAGTCATGGCGAG AAGTCATCTGCAGCCAGACG
*Vimentin*	GAGCGAGAGTGGCAGAGGAC CTTTGTCGTTGGTTAGCTGG CATATTGCTGACGTACGTCA GAGCGCCCCTAAGTTTTTAA AAGATTGCAGGGTGTTTTCG GGCCAATAGTGTCTTGGTAG
*Twist*	ACAATGACATCTAGGTCTCC CTGGTAGAGGAAGTCGATGT CAACTGTTCAGACTTCTATC CCTCTTGAGAATGCATGCAT TTTCAGTGGCTGATTGGCAC TTACCATGGGTCCTCAATAA
*IMP3*	AAGGACGAGGCCGTGACGAA TTGATCTTGGACGAGTCCAC CTTAAGAACCTACGACCGTC GGAAAACAAGGACGCACCTC AGTGCTGCACCAAGTTACTT AATTGAACCAGTCCAACCAC TCTGTACACAGCTTAACCAC CTAGTCTCGCTCAGTTTATA

RNA-ISH assay was also used for the detection of *IMP3* mRNA expression in primary and metastatic osteosarcoma tissues before systematic therapy as positive controls. After each patient was diagnosed with osteosarcoma, the biopsy tissues were taken and sliced into slides, then hybridized with the same probe and experiment protocols as CTCs identification, to explore the mRNA expression of *IMP3* in tumor tissues. In addition, we hybridized the metastatic tumor tissue of several patients before systematic therapy. Each experiment was repeated for three times and each group had four replicates.

### Detection of IMP3 protein expression

IMP3 protein expression was detected by immunohistochemistry (IHC) in normal and metastatic osteosarcoma tissues. Briefly, all the samples of tissue have been fixed in 10% formalin for 48h, and 4-μm thick sections were taken and incubated for 20 min in methanol with 10% H_2_O_2_ to block endogenous peroxidases. The sections were then incubated with the primarily monoclonal rabbit anti-human IMP3 antibody (GT226102, Gene Tec [Shanghai] Company Limited., China) at a 1:100 dilution and a biotinylated secondary antibody. The positive reaction was visualized by 3,3′-diaminobenzidine (DAB) peroxidation according to standard methods. The sections were counterstained with Mayer’s hematoxylin, dehydrated, coverslipped, and observed under an optical microscope (Olympus, Tokyo, Japan). Each experiment was repeated for three times, and each group had four replicates.

### Identification of CTC counts and EMT subtypes of patients at different time points of treatment

Serial blood samples were obtained at different time points of treatment from patients at the time of diagnosis. The patients had initially responded to neoadjuvant chemotherapy and then underwent tumor resection. After adjuvant chemotherapy, the patient developed pulmonary nodules, and radiofrequency ablation (RA) was performed for the lung metastasis. The number and classification of CTCs in blood samples were tested at different time points of treatment to explore the relationship between the change of CTCs and the therapeutic effects. Each experiment was repeated for three times and each group had four replicates.

### Statistical analysis

Statistical Product Service Solutions (SPSS) version 19.0 (IBM Corp., Armonk, NY, USA) was used to analyze the data. Data were recorded as median and range for continuous variables or as frequencies and percentages for categorical variables. The CTC counts between metastatic osteosarcoma patients and non-metastatic osteosarcoma patients were compared using nonparametric tests (Mann-Whitney *U* test). Chi-square test was used to evaluate the relationships between clinical pathological features and CTCs or *IMP3* expression in CTCs. Receiver operating characteristic (ROC) curve analysis with maximal Youden index values was applied to identify best cutoff values for different types of CTCs count for metastasis. *P*-value of < 0.05 was considered statistically significant.

## Results

### Characteristics of patients

The clinical characteristics including gender, age, tumor site, Enneking Stage of the 50 osteosarcoma patients are shown in **Table 2**. Patients consisted of 28 men and 22 women, with median age of 18.5 years (range: 8–47 years). Among them, 37 patients had tumors in the tibia or femur and 13 patients had tumors in other location. Sixty-eight percent of patients belonged to Enneking Stage IIB and 32% of patients belonged to Enneking Stage III.

**Table 2:** Clinical characteristics and CTCs test of the 50 osteosarcoma patients.

Pathological parameters		Median	Range	N	Percentage
					(%)
Total cases				50	
Gender					
	Male / Female			28/22	56/44
Age		18.5	8-47		
	≤18 years / >18 years			23/27	46/54
Tumor site		NA	NA		
	Tibia or femur / other locations			37/13	74/26
Enneking stage		NA	NA		
	IIB / III			34/16	68/32
CTCs count		4.5	0-93		
	<1 / ≥1/5ml			7/43	14/86
E type CTCs		1	0-15		
	<1 / ≥1/5ml			23/27	46/54
H type CTCs		2	0-66		
	<1 / ≥1/5ml			11/39	22/78
M type CTCs		1	0-16		
	<1 / ≥1/5ml			17/33	34/66

CTCs, circulating tumor cells; E, epithelial; H, hybrid epithelial/mesenchymal; M, mesenchymal; NA, not available; N, number of patients.

### Detection of CTCs

**Figure 2:**
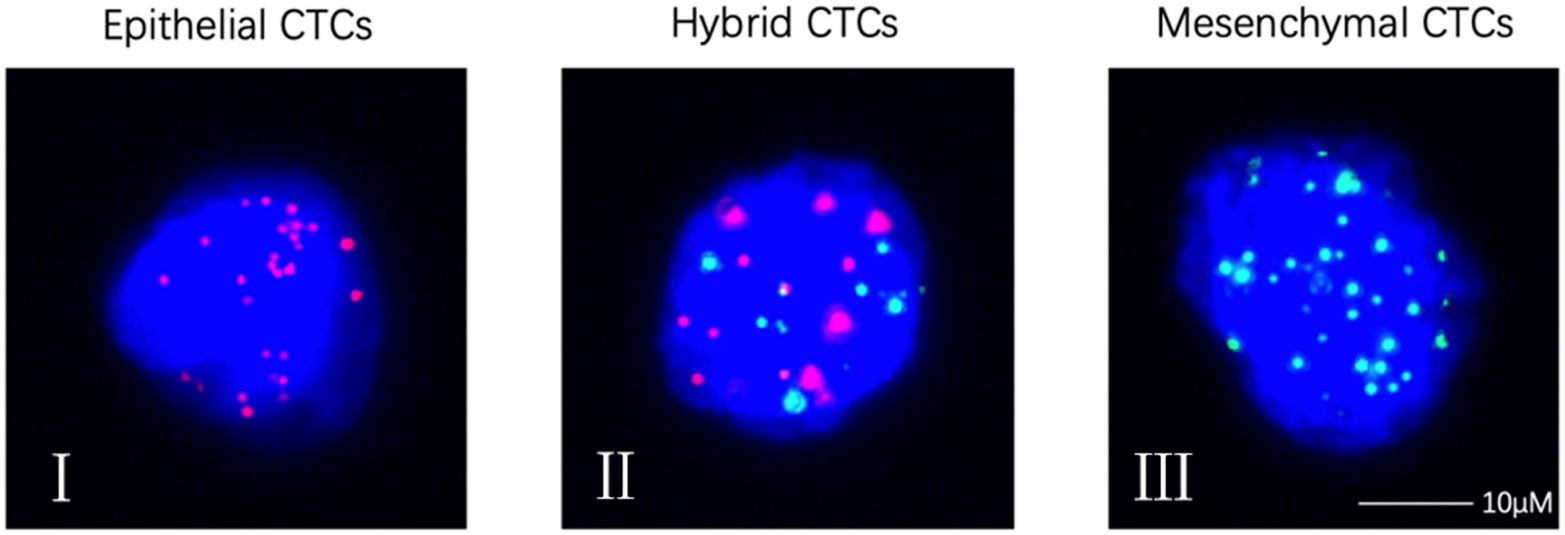
Representative images of CTCs with RNA-ISH method. (I) Epithelial CTCs stained for epithelial markers (EpCAM and CK8/18/19, red fluorescence); (II) Hybrid CTCs stained for epithelial markers (EpCAM and CK8/18/19, red fluorescence) and mesenchymal markers (vimentin and Twist, green fluorescence); (III) Mesenchymal CTCs stained for mesenchymal markers (vimentin and Twist, green fluorescence). CTCs, circulating tumor cells. Each experiment was repeated for three times and each group had four replicates.

CTCs were detected (≥ 1/5ml) in 86% (43/50) of the osteosarcoma patients (**Table 2**). The median number of CTCs was 4.5 (range: 0–93) in 5 ml peripheral blood sample of all 50 patients. Using the Canpatrol CTC detection platform, all the separated CTCs were divided into three EMT types (E type, H type, and M type) with different labeled mRNA probes. As shown in **Figure 1**, I represented E-type cells, II represented H-type cells, and III represented M-type cells, respectively (**Figure 2**). E-type, H-type, and M-type CTCs were detected in about 54, 78, and 66% osteosarcoma patients, respectively, and the median number of each type CTCs in 5 ml of peripheral blood was 1 (range: 0–15), 2 (range: 0–66), and 1 (range: 0–16) (**Table 2**). Notably, no CTC was detected in peripheral blood samples from five healthy donors.

### Correlation between CTCs and clinical features

**Figure 3:**
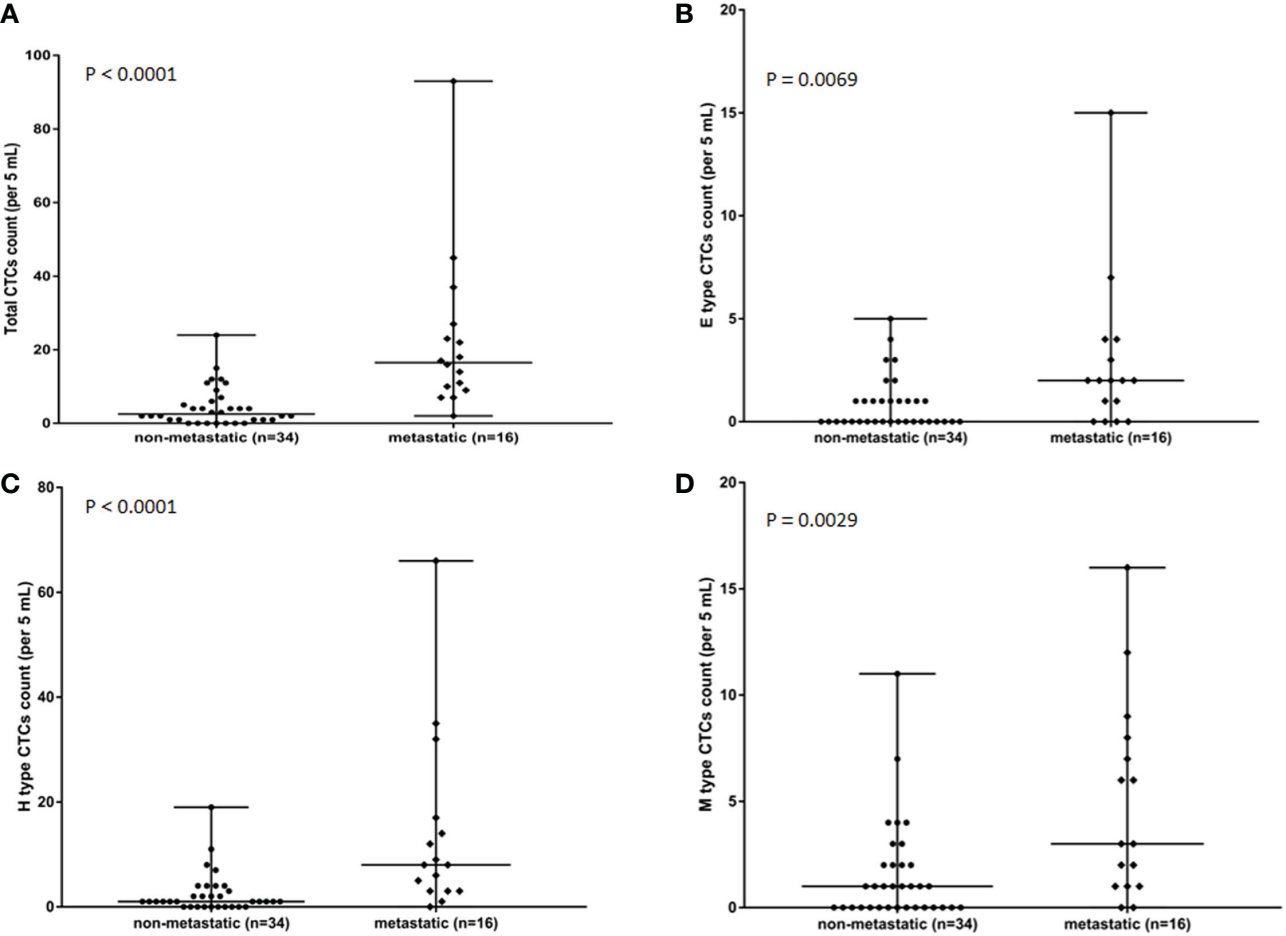
Associations in total CTC number **(A)**, E-type CTC number **(B)**, H-type CTCs number **(C)**, and M-type CTCs numbers **(D)** in metastatic osteosarcoma patients (*n* = 16) and non-metastatic osteosarcoma (*n* = 34) patients. *P* value was analyzed with Mann-Whitney *U* test. Each experiment was repeated for three times, and each group had four replicates.

The relationship between CTCs and clinical features in 50 osteosarcoma patients is shown in **Table 3**. CTCs were identified in peripheral blood of 27 of 34 non-metastatic (Enneking Stage IIB) osteosarcoma patients and all 16 metastatic (Enneking Stage III) osteosarcoma patients. The CTC counts, especially the total CTC counts, and the H-type CTC counts significantly differed between Enneking Stage IIB and Stage III patients (*P* < 0.001). No significant differences were observed between the CTC count or type and other clinicopathological features, such as gender, age, or tumor location (*P* > 0.05). It was shown that the total CTC count (**Figure 3A**), E-type CTC count (**Figure 3B**), H-type CTC count (**Figure 3C**), M-type CTC count (**Figure 3D**) were significantly higher in metastatic osteosarcoma patients compared with that in non-metastatic osteosarcoma patients (all *P* < 0.05). Interestingly, compared with M-type CTCs, the number of total CTCs and H-type CTCs differed more between metastatic and non-metastatic patients.

**Table 3:** Association between CTCs and clinical variables in 50 osteosarcoma patients.

	N	Total CTCs		E type CTCs		H type CTCs		M type CTCs		IMP3 (+) CTCs	
		Median	*P*	Median	*P*	Median	*P*	Median	*P*	Median	*P*
Gender											
Male	28	2.5 (0-37)	0.154	0.5 (0-4)	0.779	1 (0-32)	0.174	0 (0-8)	0.918	1 (0-23)	0.106
Female	22	10 (0-93)		1 (0-15)		4 (0-66)		1 (0-16)		5 (0-61)	
Age											
≤18 years	23	4 (0-45)	0.526	1 (0-7)	0.749	2 (0-35)	0.370	1 (0-9)	0.889	1 (0-37)	0.558
>18 years	27	7 (0-93)		1 (0-15)		3 (0-66)		1 (0-16)		3 (0-61)	
Tumor location											
Tibia or femur	37	4 (0-93)	0.641	0 (0-15)	0.489	2 (0-66)	0.911	1 (0-12)	0.307	2 (0-61)	0.604
Other locations	13	7 (0-45)		1 (0-7)		3 (0-35)		2 (0-16)		3 (0-37)	
Enneking Stage											
IIB	34	3 (0-24)	0.000	0 (0-5)	0.007	1 (0-19)	0.000	1 (0-11)	0.004	1 (0-14)	0.000
III	16	16.5 (0-93)		2 (0-15)		8 (0-66)		3 (0-16)		9.5 (0-61)	

CTCs, circulating tumor cells; E, epithelial; H, hybrid epithelial/mesenchymal; M, mesenchymal; P<0.05, with statistically significant difference.

### The expression of *IMP3* in CTCs and tumor tissues

**Figure 4:**
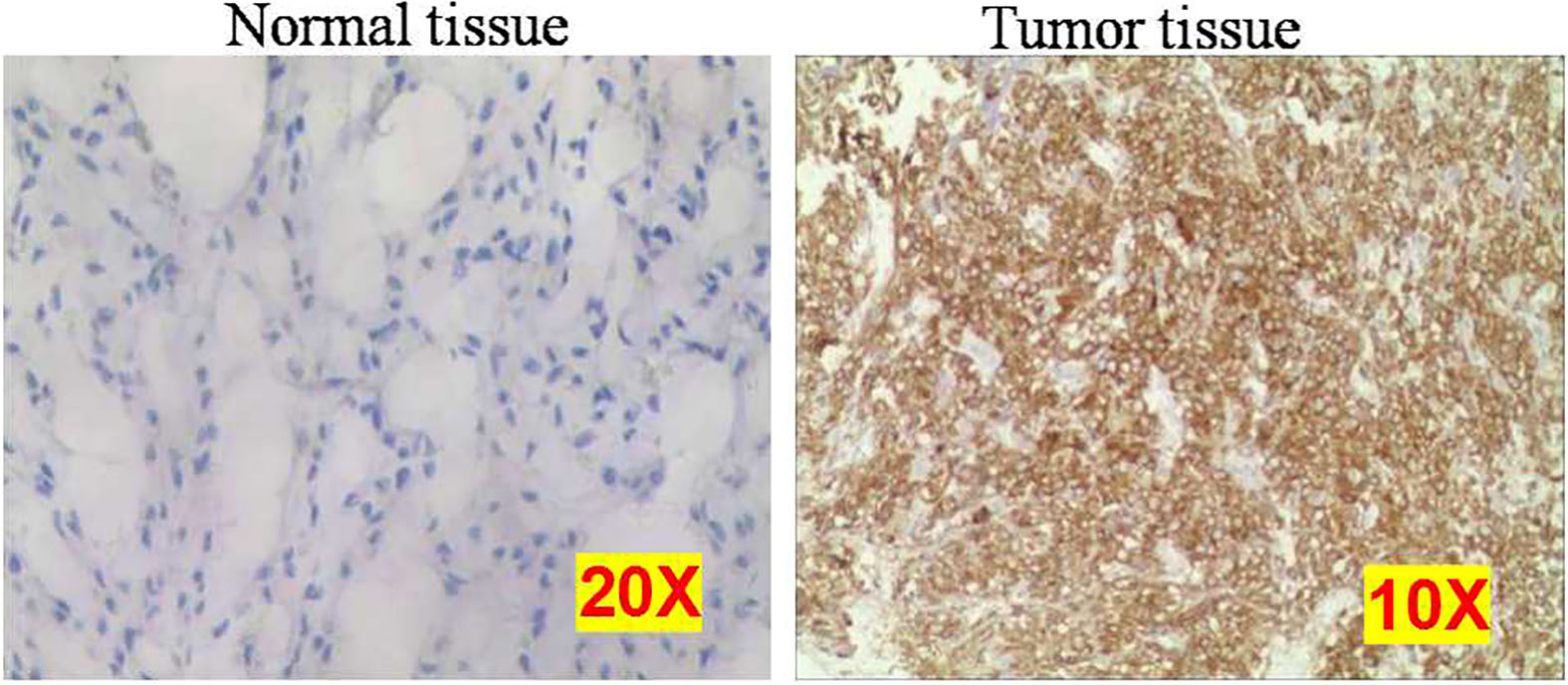
Representative images of normal and osteosarcoma tissues using immunohistochemistry. Each experiment was repeated for three times, and each group had four replicates.

The mRNA expression of *IMP3* was detected by RNA-ISH assay (**Table 4**), and IMP3 protein expression was detected by IHC (**Figure 4**) in normal and osteosarcoma tissues. The results showed that the *IMP3* gene was expressed in 72% (36/50) of the overall patients and 83.7% (36/43) of CTCs positive patients. The median number of *IMP3*-positive CTCs was 2 in 5 ml of blood samples of patients. Further study showed that the expression rates of *IMP3* in different types of CTCs were different: 38.4% (28/73) in the E-type CTCs, 65.6% (200/305) in the H-type CTCs, and 62.0% (80/129) in the M-type CTCs. The difference of *IMP3* expression among them was statistically significant (*P* < 0.001; **Table 4**). Compared with the control, IMP3 protein expression increased in osteosarcoma tissues (**Figure 4**).

**Table 4:** The expression rates of the *IMP3* gene in different types of CTCs.

Types of CTCs	NO. of CTCs	Positive for *IMP3*		Negative for *IMP3*		χ^2^ test	
		N	Percentage, %	N	Percentage, %	χ^2^	*P*
E type CTCs	73	28	38.4	45	61.6	18.416	< 0.001
H type CTCs	305	200	65.6	105	34.4		
M type CTCs	129	80	62.0	49	38.0		

CTCs, circulating tumor cells; E, epithelial; H, hybrid epithelial/mesenchymal; M, mesenchymal; NO., number; N, number of patients.

**Figure 5:**
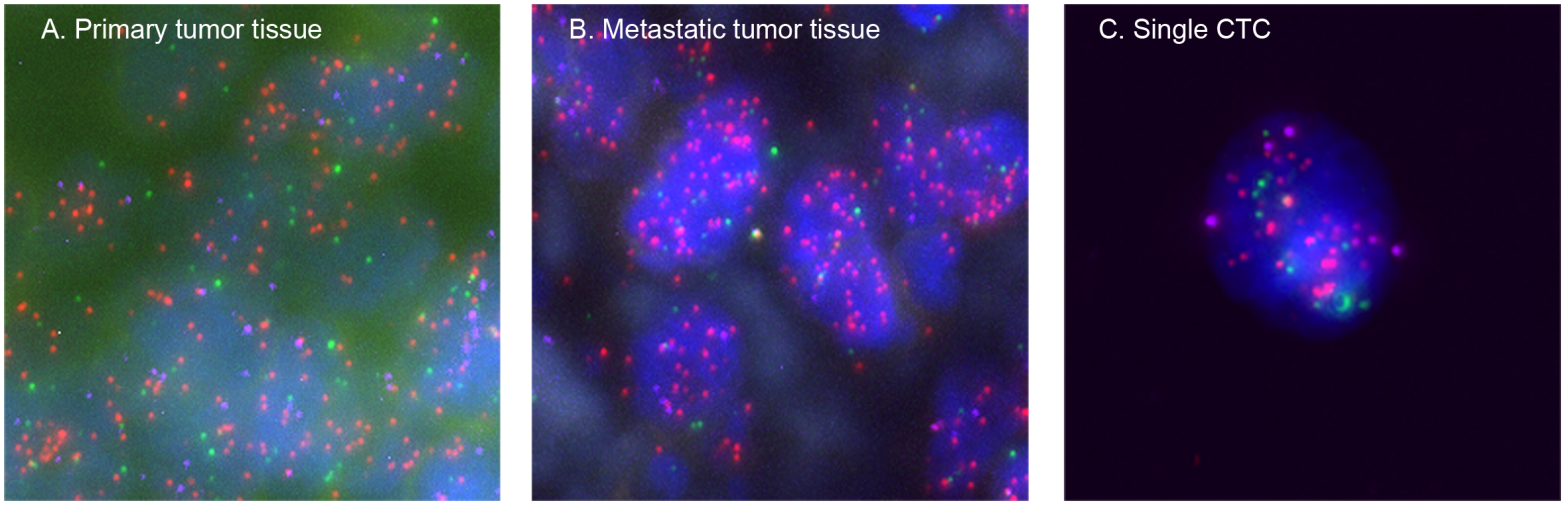
Representative images of the osteosarcoma tissues and CTCs based on RNA-ISH staining of epithelial (EpCAM and CK8/18/19, red fluorescence), mesenchymal (vimentin and Twist, green fluorescence), and IMP3 (purple fluorescence) markers. **(A)** From a primary osteosarcoma tissue, **(B)** from a metastatic osteosarcoma tissue, and **(C)** from a single CTC. Each experiment was repeated for three times, and each group had four replicates.

To verify that these CTCs were from the primary or metastatic osteosarcoma tissues and not from other tissues, all patients’ primary tumor tissues and several patients’ metastatic tumor tissues were analyzed using the same probes as in CTCs. The same fluorescence signals were detected in these tissues, confirming that the CTCs originated from the primary or metastatic tumors (**Figure 5**).

### *IMP3* expression correlates with Enneking Stage of osteosarcoma

The relationship between the expression of *IMP3* in CTCs and the clinical characteristics of patients was also analyzed (**Table 5**). Because the median number of *IMP3*-positive CTCs was 2 in 5 ml of blood samples, the patients were divided into two groups: > 2 *IMP3*-positive CTCs per 5 ml of blood sample and ≤ 2 *IMP3* positive CTCs per 5 ml of blood sample. It was found that the presence of > 2 *IMP3*-positive CTCs was significantly associated with later Enneking Stage *versus* ≤ 2 *IMP3*-positive CTCs (*P* < 0.001), but there were no differences in other clinicopathological features (*P* > 0.05).

**Table 5:** Relationship between *IMP3* expression in CTCs and clinical pathological features.

Pathological parameters		N	*IMP3* expression		χ^2^ test	
			≤2 *IMP3* positive CTCs	>2 *IMP3* positive CTCs	χ^2^	*P*
Total cases		50	28	22		
Gender	Male	28	19	9	3.631	0.086
	Female	22	9	13		
Age	≤ 18	23	15	8	1.469	0.264
	>18	27	13	14		
Tumor location	Tibia or femur	37	22	15	0.691	0.520
	other	13	6	7		
Enneking Stage	IIB	34	26	8	18.070	< 0.001
	III	16	2	14		

CTCs, circulating tumor cells; N, number of patients.

**Figure 6:**
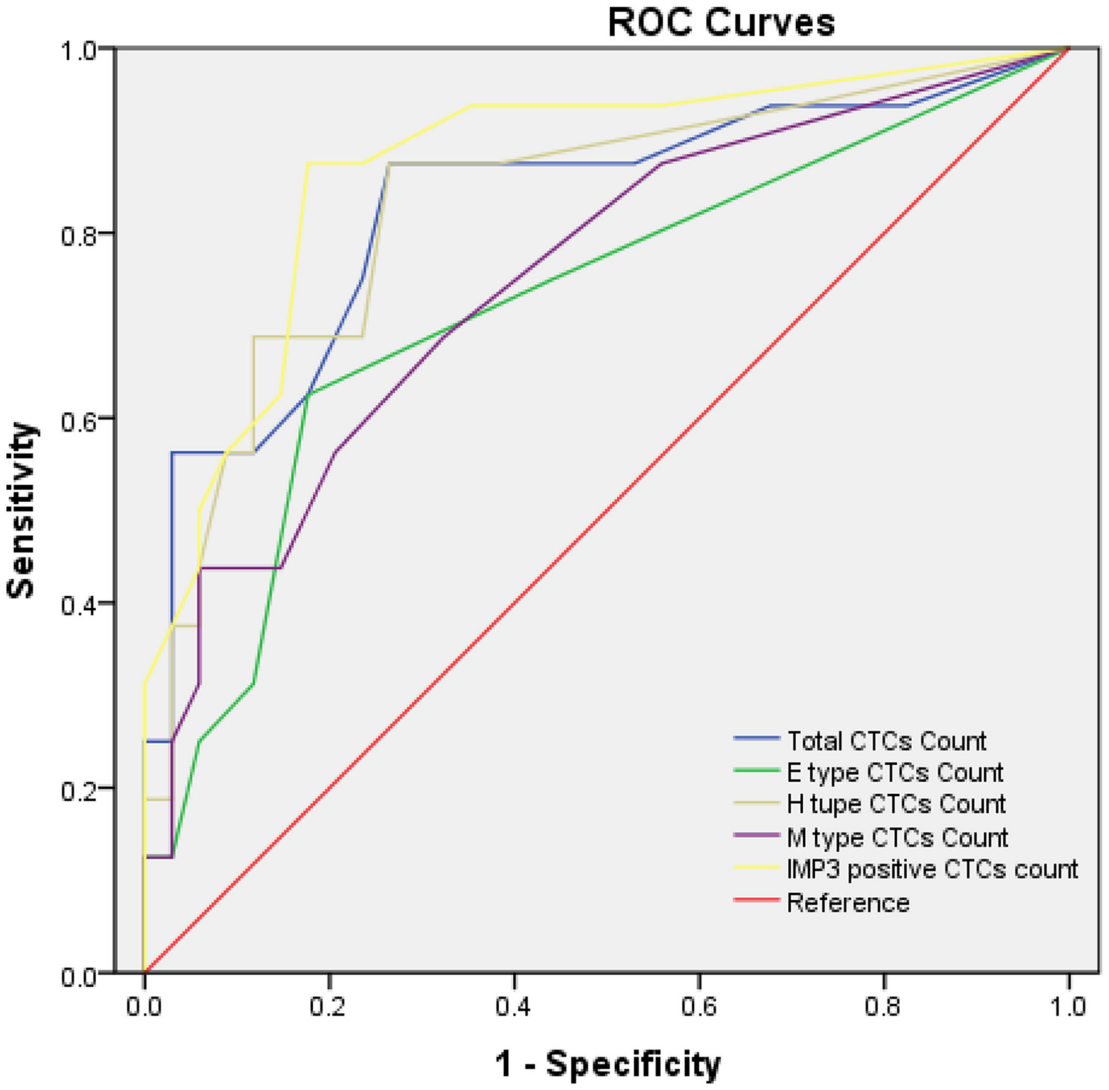
ROC curves for total CTC count and IMP3-positive CTC count. The best cutoff value was 6.5 cells/5ml of peripheral blood for total CTC count and four cells/5ml of peripheral blood for IMP3-positive CTC count according to the maximization of Younden index from the ROC curve analysis. Each experiment was repeated for three times, and each group had six replicates.

We also analyzed whether different types of CTCs and *IMP3*-positive CTCs could be used as auxiliary diagnostic biomarkers for osteosarcoma metastasis. ROC curve analysis showed the area under the curve values of 0.724 (95% CI, 0.564–0.884; *P* = 0.011) for E-type CTC count, 0.751 (95% CI, 0.602–0.900; *P* = 0.005) for M-type CTC count, 0.830 (95% CI, 0.696–0.964; *P* < 0.001) for total CTC count, 0.833 (95% CI, 0.704–0.962; *P* < 0.001) for H-type CTCs count, and 0.873 (95% CI, 0.762–0.984; *P* < 0.001) for *IMP3*-positive CTCs count, respectively (**Figure 6**). The *IMP3*-positive CTCs count presented the largest area under the curve (0.873 [95% CI, 0.762–0.984; *P* < 0.001]), with the cutoff value of four cells/5ml, with a sensitivity of 87.5% and a specificity of 82.4%. The best cutoff was 6.5 cells/5ml for total CTC count, 1.5 cells/5ml for E-type CTC count, 5.5 cells/5ml for H-type CTCs count, and five cells/5ml for M-type CTCs count according to maximization of the Younden index (total CTC count: sensitivity = 87.5% and specificity = 73.5%; E-type CTC count: sensitivity = 62.5% and specificity = 82.5%; H-type CTC count: sensitivity = 68.8% and specificity = 88.2%; M-type CTC count: sensitivity = 43.8% and specificity = 94.1%). Compared with other phenotypic CTCs, M-type CTCs and H-type CTCs had better specificity.

### Dynamic changes of CTC counts and EMT subtypes following treatment

**Figure 7:**
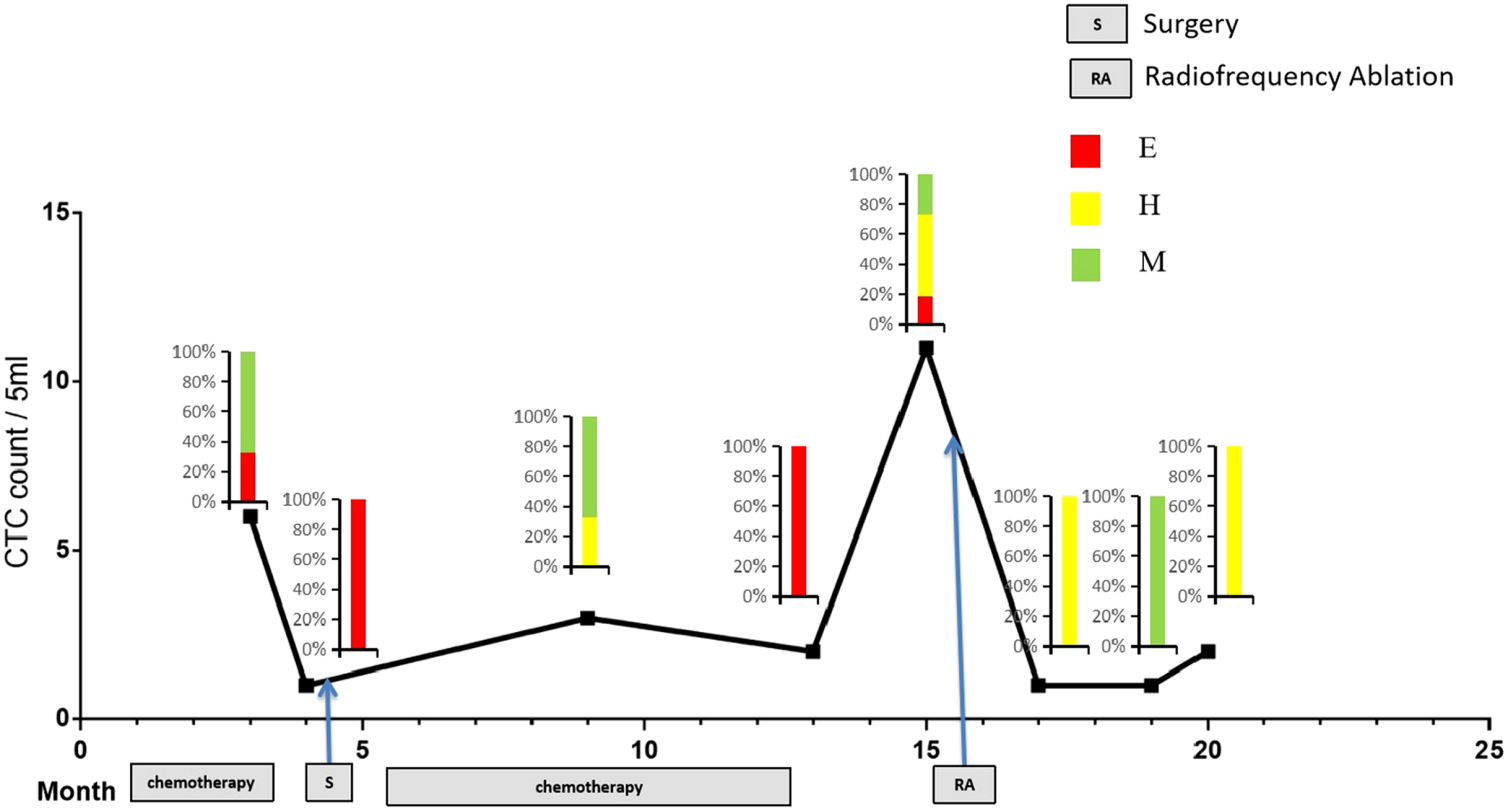
Longitudinal monitoring of ETM CTCs in patients. Plot of CTC counts per 5 ml of blood based on Canpatrol platform in a patient who was serially sampled during treatment with neoadjuvant chemotherapy, followed by surgery and adjuvant chemotherapy. Color-coded quantitation of EMT features is shown above each time point. Each experiment was repeated for three times, and each group had four replicates.

To better study the relationship of CTCs in predicting treatment response, longitudinal CTC monitoring was performed in patients who underwent neoadjuvant chemotherapy, surgery, and adjuvant chemotherapy. As shown in **Figure 6**, CTC count was decreased after neoadjuvant chemotherapy, especially the proportion of M-type CTCs, along with tumor shrinkage. Subsequently, surgical treatment was performed on the patients, followed by adjuvant chemotherapy, and the CTC count stabilized for about 8 months. However, 3 months after the end of systemic adjuvant chemotherapy, the CTC count increased dramatically from two to 11, together with proportion of H-type CTCs and M-type CTCs. Meanwhile, the patient exhibited radiographically progress, one pulmonary nodule detected on CT scan, suspected lung metastasis. Therefore, RA was performed on the patients, and the CTC count was decreased to one after 1 month (**Figure 7**).

## Discussion

Metastasis is an important clinical problem of osteosarcoma. Even if surgery is combined with chemotherapy and other comprehensive treatments, 40–50% of patients still have lung metastasis, which seriously affects the prognosis of patients (28, 29). At present, the evaluation of the condition of patients, the treatment effects, and the diagnosis of metastasis and recurrence mainly rely on traditional imaging examinations (30, 31). It can be seen that the existing clinical evaluation methods are inaccurate in evaluating the prognosis of patients with osteosarcoma (32–34). CTCs, which shed from primary or metastatic tumors, carry biological information of tumor metastasis. Compared with traditional tissue biopsy, CTC detection is non-invasive and can overcome tumor heterogeneity to provide more comprehensive information of tumor (35). Thus, investigating phenotype of CTCs and target biomarker expression in CTCs plays important role in understanding of tumor information, which is the basis of tumor individualized treatment (36, 37). However, many techniques are only based on epithelial markers to detect and isolate CTCs, which is likely to miss the CTC subsets undergoing EMT, especially for tumors of interstitial origin such as osteosarcoma. In addition, osteosarcoma is a tumor that metastasizes mainly *via* blood way, and the exploration of CTCs in the peripheral blood is of great importance to clinical management. Based on the isolation and detection of CTCs, this study creatively detected the expression of *IMP3*-gene expression, and the CTC count and proved that the *IMP3*-positive CTCs and H/M-type CTCs are related to the metastasis of osteosarcoma,

CTC count is proved to predict poor prognostic in metastatic breast cancer, with the CellSearch CTC enrichment technique (38). However, EMT may lead to an underestimation of CTC number, since its widely reported prerequisite for metastasis (39, 40). This study investigated the relationship between the number or classification of CTCs in peripheral blood and the clinical characteristics of patients, and found that CTCs were detected in 43 of 50 osteosarcomas patients, covering both Enneking IIB and Enneking III, with a positive rate of 86%. The CTCs count is significantly correlated with Enneking Stage but not with other clinical features, such as age and gender. The results are consistent with those of previous studies (17–20). These results indicated that the CTCs count may be used as a supplementary method for imaging examinations to assist physicians in analyzing patient conditions. Furthermore, the dynamic changes of CTCs count and phenotype are related to treatment response and disease progression. When the patient responded to treatment, the CTC count decreased; however, the number of CTCs increased when disease progression. These results suggest that, after tumor resection or systemic chemotherapy, CTC detection can be used as a real-time and noninvasive method to evaluate the therapeutic efficacy or monitor tumor progression.

It has been reported that the ratio of E-type CTCs, M-type CTCs, and H-type CTCs can be used to monitor the therapeutic effect. Yu and his colleagues found a subset of CTCs with mixed E/M phenotypes and observed an increase in the number and proportion of M phenotypes at the stage of disease progression (16), further indicating that M-type CTCs are involved in the metastasis process. Previous studies also suggested that the number and proportion of M-type CTCs may be more appropriate for predicting drug resistance and evaluating prognosis than total CTC count (41, 42). However, there are no similar reports in osteosarcoma. Here, the Canpatrol system was utilized to isolate and identify CTCs in osteosarcoma patients, which can classify CTCs into E, H, and M phenotypes. We dynamically monitored the change of CTCs in patients during the whole course of treatment and showed that the tumor size decreases after neoadjuvant treatment, accompanied by the decrease of CTCs count and the switch of CTCs phenotype. Three months after the end of adjuvant chemotherapy, a pulmonary nodule was detected by CT scan, and the number of CTCs increased significantly. After RA treatment, the number of CTCs decreased again. This suggests that the CTCs count can reflect tumor load in a certain extent. In addition, the CTCs, as the seed of metastasis, are likely to exist in the peripheral blood before the occurrence of lung lesion, and the tumor may have metastasized before the presence of CT scan-visible lesion in the patient’s lung. At the end of postoperative chemotherapy, the lung lesion was no longer inhibited by chemotherapy drugs, so they increased to the extent of CT scan visible within 3 months. Therefore, our study provides a new perspective for the dynamic monitoring of tumor and some basis for early intervention before the occurrence of imaging visible metastatic lesions.

This study not only verified the important clinical significance of CTCs count in osteosarcoma but also showed that CTCs count combined with its molecular characteristics can more accurately characterize the prognosis of patients. *IMP3* is a member of the IMP family, which is low expressed or at undetectable protein levels in normal adult tissues (43–45). On the contrary, *IMP3* protein expression is strongly upregulated in some malignant tumors (46–48). It was found that high expressions of *IMP3* are positively correlated with stage and pulmonary metastasis of osteosarcoma and can be applied as a potential indicator of the malignant degree of osteosarcoma and the prognosis of patients (26). This study made a breakthrough on the relationship between the EMT of CTCs and expression of *IMP3* gene and found that the positive rate of *IMP3* expression in E-type CTCs, H-type CTCs, and M-type CTCs are 38.4, 65.6, and 62.9%, respectively. In addition, compared with the expression level of *IMP3* in CTCs of patients with stage IIB, the expression levels of *IMP3* are significantly higher in CTCs of patients with stage III. These findings suggest that the expression of *IMP3* may promote the process of EMT and indicate the distant metastases and poor prognosis of patients. Furthermore, ROC analysis showed that *IMP3*-positive CTC count has the best performance for diagnostic metastasis, with the largest area under the curve of 0.873 and cutoff value of four cells/5ml peripheral blood (sensitivity = 87.5% and specificity = 82.4%). It is noteworthy that the most specific marker for the diagnosis of metastasis is M-type CTC, followed by H-type CTC, with specificity of 94.1 and 88.2%, respectively, with the cutoff value of five cells/5ml and 5.5 cells per 5 ml of peripheral blood. These results suggest that molecular characterization of CTCs is a more accurate assessment of patient prognosis than CTC counts alone, particularly in CTCs that have undergone EMT and those with positive *IMP3* expression. The study may provide a new means for comprehensive understanding of osteosarcoma, and the expression of *IMP3* gene in CTCs may be a potential new marker for predicting the prognosis of osteosarcoma and inhibition of osteosarcoma metastasis.

There are some limitations of this study. First, because the Canpatrol system is based on the size of tumor cells, some small CTCs may be missed. Second, due to the small number of patients in our study, the results should be interpreted with caution. Furthermore, CTCs were detected in all patients at baseline, but only a few patients underwent dynamic monitoring of CTCs. Therefore, more evidence is needed to support the important role of CTCs dynamic monitoring in the treatment management of tumor patients. Third, each type of CTCs was not cultured *in vitro*, and the biological characteristics were not identified and compared, including cell proliferation, migration and invasion ability, and *IMP3* expression. Fourth, the tumor formation and capability of metastasis of each type CTCs *in vivo* should be tested by animal experiment. Fifth, *IMP3* overexpression and knockdown CTCs could be established, and the migration and invasion ability should be also detected *in vitro*. We should provide information about that in further studies.

## Conclusions

In summary, the positivity of CTCs in the peripheral blood of patients with osteosarcoma is very high, and the number and classification of CTCs are correlated with the Enneking Stage of the tumor, with significantly more CTCs in patients with Enneking Stage III compared with those with Enneking Stage IIB. The *IMP3* mRNA expression in CTCs is higher in advanced patients and more in CTCs with EMT. ROC analysis shows that *IMP3*-positive CTC count performs optimally in aiding the diagnosis of metastasis, and ≥ 4 *IMP3*-positive CTCs per 5 ml of peripheral blood is a predictor of metastasis. Furthermore, dynamic monitoring of the number and phenotype of CTCs allows assessment of disease progression in patients and may guide individualized treatment in the near future. Thus, dynamic monitoring CTC count in patients with osteosarcoma and the application of molecular methods for CTCs may be of great clinical importance for the timely detection of recurrence or metastasis.

## Data availability statement

The original contributions presented in the study are included in the article/supplementary material. Further inquiries can be directed to the corresponding authors.

## Ethics statement

The studies involving human participants were reviewed and approved by The Third Affiliated Hospital of Southern Medical University Clinical Trial Ethics Committee. Written informed consent to participate in this study was provided by the participants’ legal guardian/next of kin.

## Author contributions

HL and DJ: conception and design. SDa and XS: development of methodology. SDa, QW, SDu, and CH: acquisition of data. SDa and XS: analysis and interpretation of data and writing, review, and/or revision of the manuscript. HL and DJ: study supervision. All authors contributed to the article and approved the submitted version.
